# A cello concert in Brazilian lands: the life, art, and disease of Jacqueline du Pré

**DOI:** 10.1055/s-0045-1811726

**Published:** 2025-09-19

**Authors:** Diogo Haddad-Santos, Filipi Fim Andreão, Filipe Virgilio Ribeiro, Karlos Daniell Araújo dos Santos, Fernanda Herculano, Guilherme Sciascia do Olival, Rafael Paterno Dias Carneiro

**Affiliations:** 1 Santa Casa de São Paulo, Faculdade de Ciências Médicas, Departamento de Neurologia, São Paulo SP, Brazil.; 2 Hospital Alemão Oswaldo Cruz, São Paulo SP, Brazil.; 3 Universidade Federal do Rio de Janeiro, Rio de Janeiro RJ, Brazil.; 4 Centro Universitário Barão de Mauá, Faculdade de Medicina, Ribeirão Preto SP, Brazil.; 5 Universidade Federal de Roraima, Boa Vista RR, Brazil.

**Keywords:** Multiple Sclerosis, Art, Culture, Health, Awareness

## Abstract

The present paper explores the extraordinary life of cellist Jacqueline du Pré, her remarkable contribution to music, and her battle with multiple sclerosis (MS). Beyond her artistic impact, we discuss how her story influenced the creation of the Brazilian Multiple Sclerosis Association (Associação Brasileira de Esclerose Múltipla, ABEM, in Portuguese) and its cultural significance in Brazil, particularly through the play *Duet for One* , by Tom Kempinski. The study reflects on the role of art as a powerful tool to raise awareness and contribute to the understanding of diseases.

## INTRODUCTION

**Figure 1:**
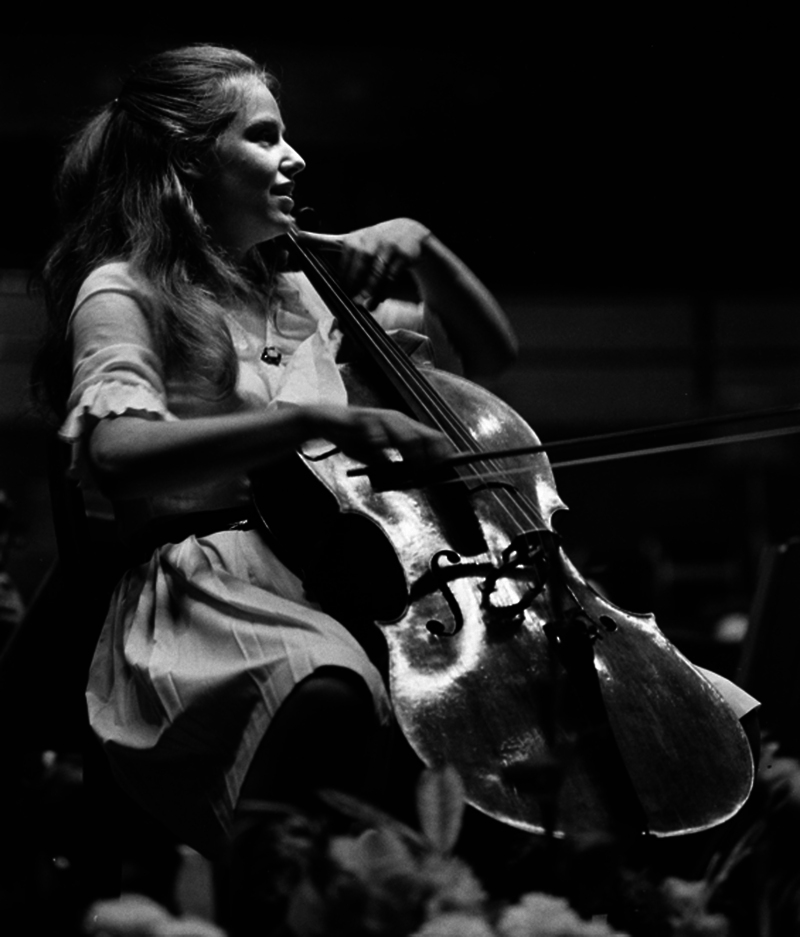
Picture of Jacqueline Mary du Pré. Source: Chicago Symphony Orchestra Archives (2014).

Jacqueline du Pré ( Figure 1 ) was a transcendent force in classical music, known for her emotionally-charged interpretations and impeccable technique. However, her career was tragically cut short by multiple sclerosis (MS), which was diagnosed in 1973. The present paper celebrates her artistic achievements and highlights how her journey with MS contributed to the visibility and understanding of the disease.

## LIFE AND ART

Born in Oxford, England, Jacqueline was raised in a musically-inclined family. Her mother was a pianist, and her father had a deep love for classical music. Immersed in this environment, Jacqueline developed an early affinity for the cello, beginning lessons at the age of 5 years. Her extraordinary talent led her to the Guildhall School of Music and Drama, where she studied under William Pleeth. At 15, she won the Queen's Prize, marking the start of a distinguished career. At 16, she debuted professionally in London, performing Elgar's “Cello Concerto in E minor” with the London Symphony Orchestra. This piece became synonymous with her name and cemented her place as a prodigious talent. 1 2

By 18, Jacqueline was a soloist with the BBC Symphony Orchestra, an extraordinary feat that reflected her charismatic stage presence. Her renown grew beyond England, leading to international tours, including a highly-acclaimed United States tour. She gained a reputation as a deeply-emotional performer, capable of conveying the essence of complex compositions with remarkable intimacy. 1

Jacqueline was also an accomplished chamber musician, collaborating with renowned artists such as Daniel Barenboim, Pinchas Zukerman, and Itzhak Perlman. These collaborations resulted in highly-regarded recordings that enriched the classical music repertoire. Among them, her recording of Elgar's “Cello Concerto”, conducted by Sir John Barbirolli, remains one of the greatest interpretations of the work. Her meteoric rise was defined by performances that combined flawless technique with deep emotional resonance, securing her status as one of the most remarkable cellists of the twentieth century.

## THE BATTLE AGAINST MULTIPLE SCLEROSIS

Jacqueline du Pré's brilliant trajectory was tragically altered by MS, a chronic autoimmune disease that affects the central nervous system. The first signs of the disease appeared subtly, with a numbness in her fingertips—a devastating premonition for a cellist. Over time, her physical changes became more pronounced, affecting her ability to perform. The progression of MS was exacerbated by the side effects of the treatments, particularly the one with corticosteroids, which caused weight gain and altered her appearance. 3

Her final performances revealed the full extent of her struggle. While still filled with emotion, they were marked by visible difficulties in coordination and strength. The fluidity and control that once defined her playing had become compromised. Yet, her determination to perform despite the challenges was a testament to her resilience. Emotionally, she oscillated between denial and acceptance, eventually confiding in close friends and family. 1

Jacqueline and her husband, Daniel Barenboim, sought treatment in various countries, including the Soviet Union and the United States. However, at the time, the available treatments could do little to halt the disease's progression. Her treatment with corticosteroids led to visible signs of Cushing's syndrome, including facial swelling and a curved posture. These changes were evident in interviews and public appearances in the 1970s and 1980s. 2

Despite her suffering, Jacqueline du Pré transformed her battle into a powerful legacy of resilience. Her story highlighted both the fragility of the human body and the strength of the spirit, inspiring not only musicians but all those who face adversity. She passed away in 1987. Notably, 6 years after her passing, significant advancements in MS treatment emerged. In 1993, a clinical trial 4 demonstrated that interferon beta-1b (IFNβ-1b) improved conditions in nearly 30% of patients with relapsing-remitting MS (RRMS), establishing it as the first effective therapy to modify the disease course. Subsequent studies 5 6 emphasized the importance of early treatment initiation.

## CULTURAL IMPACT AND LEGACY

**Figure 2:**
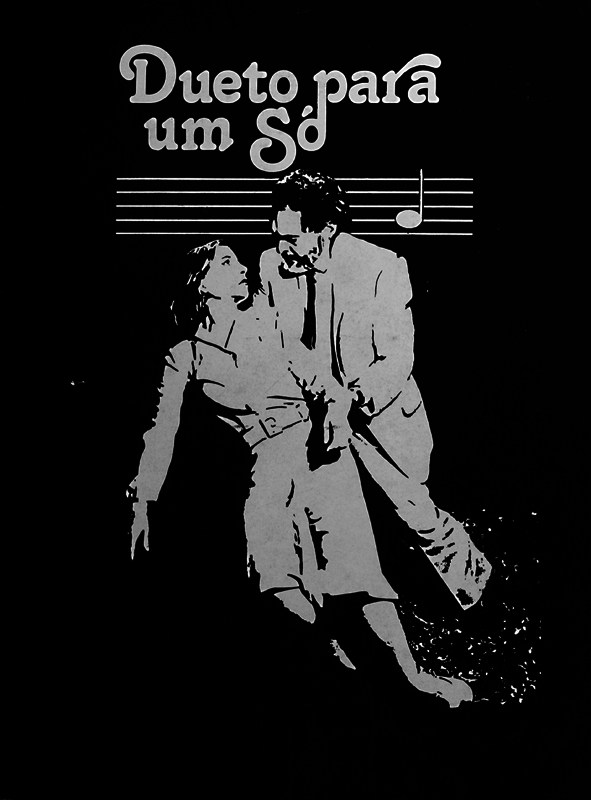
Booklet of the play *Duet for One* , by Tom Kempinski.

Beyond her unforgettable performances, Jacqueline du Pré profoundly influenced the arts and MS awareness. The play *Duet for One* , by Tom Kempinski ( Figure 2 ), is a remarkable artistic expression of this legacy. Inspired by du Pré's battle with MS, the play provides an emotionally-charged perspective on the challenges faced by those with chronic illnesses, particularly their impact on identity and relationships.

In Brazil, *Duet for One* premiered in 1984 at the Ruth Escobar Theater, in the city of São Paulo, starring actors Othon Bastos and Martha Overbeck. The play not only captivated audiences but also played a crucial role in raising awareness about MS. Informational pamphlets on the disease, written by Professor Wilson Luiz Sanvito, were distributed to theatergoers, making the play a significant moment for MS awareness in Brazil.

An unexpected outcome of the play was the connection formed among audience members affected by MS. Those using canes or wheelchairs found in each other a sense of solidarity. This led to the foundation of the Brazilian Multiple Sclerosis Association (Associação Brasileira de Esclerose Múltipla, ABEM, in Portuguese) by Ana Maria Almeida Amarante Levy and Dr. Renato Basile. 1 2

Initially, ABEM meetings were held in Ana Maria Levy's apartment. As the group expanded, a permanent space was established at Lar Escola São Francisco, and later, additional chapters were created in Rio de Janeiro and other cities. Organizations such as ABEM play an essential role in providing resources, support, and advocacy for MS patients, increasing the visibility of chronic illnesses, and fostering a more empathetic and informed society. 7

In conclusion, Jacqueline du Pré's legacy extends beyond her invaluable contributions to classical music. Her story has inspired the creation of significant platforms for discussion and confrontation of MS, fostering greater understanding and support for those affected worldwide. Through art, science, and solidarity, the fight against multiple sclerosis continues, echoing du Pré's resilient spirit. By intertwining her life and illness, du Pré and her representation demonstrate the power of art to transcend the personal, leaving a deep and lasting impact on society.
